# *CHML* is an NRF2 target gene that regulates mTOR function

**DOI:** 10.1002/1878-0261.13194

**Published:** 2022-02-28

**Authors:** Matthew Dodson, Wujing Dai, Annadurai Anandhan, Cody J. Schmidlin, Pengfei Liu, Nathan C. Wilson, Yongyi Wei, Naoya Kitamura, James J. Galligan, Aikseng Ooi, Eli Chapman, Donna D. Zhang

**Affiliations:** ^1^ Department of Pharmacology and Toxicology College of Pharmacy University of Arizona Tucson AZ USA; ^2^ University of Arizona Cancer Center University of Arizona Tucson AZ USA

**Keywords:** *CHML/REP2*, mTOR, NRF2, NSCLC

## Abstract

The transcription factor nuclear factor erythroid 2‐related factor 2 (NRF2) is often highly expressed in non‐small cell lung cancer (NSCLC). Through its target genes, NRF2 enhances cancer progression and chemo/radioresistance, leading to a poorer prognosis in patients with high NRF2 expression. In this study, we identified CHM‐like Rab escort protein (*CHML*; encoding Rep2) as an NRF2 target gene with an antioxidant response element (ARE) in its promoter region (–1622 to –1612). Analysis of patient data curated by The Cancer Genome Atlas (TCGA) and Oncomine databases revealed that *CHML* mRNA expression was elevated in lung adenocarcinoma (LUAD) patient tumor tissues and correlated with decreased patient survival. Immunohistochemistry (IHC) analysis of normal versus lung cancer patient tissues revealed that Rep2 protein levels were higher in lung tumors compared with normal tissue, which also correlated with increased levels of NRF2. Importantly, siRNA‐mediated knockdown of *CHML*/Rep2 in A549 NSCLC cells decreased their ability to proliferate. Mechanistically, Rep2 mediates mTOR function, as loss of Rep2 inhibited, whereas overexpression enhanced, mTOR translocation and activation at the lysosome. Our findings identify a novel NRF2–Rep2‐dependent regulation of mTOR function.

## Graphical abstract

Constitutive activation of NRF2 and its downstream targets is a key driver of non‐small cell lung cancer (NSCLC) progression. Here, we identified CHM‐like Rab escort protein (*CHML*/Rep2) as a transcriptional target of NRF2 that mediates mTOR translocation to the lysosome and initiation of protein translation. Thus, the NRF2‐CHML‐mTOR axis could represent a key mediator of NSCLC growth and survival.

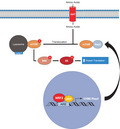

**Abbreviations**
ARE: antioxidant response element
Bru: Brusatol
*CHML*/Rep2: CHM like Rab escort protein
GGTase: geranylgeranyl transferase
KEAP1: Kelch‐like ECH associated protein 1
LC3: microtubule‐associated protein 1A/1B‐light chain 3
LUAD: lung adenocarcinoma
mTOR: mechanistic target of rapamycin
NRF2: nuclear factor erythroid 2‐related factor 2
NSCLC: non‐small cell lung cancer
R: L‐arginine
S6: ribosomal protein S6
S6K: S6 kinase
SF: sulforaphane
SQSTM1/p62: sequestosome 1
TCGA: the cancer genome atlas

## 1 Introduction

Despite decades of effort, many cancers remain difficult to treat, particularly when discovered in the later stages. This is due, at least in part, to the ability of cancer cells to alter their metabolic programming to adapt to harsh conditions, such as the demands of rapid growth, an unfavorable tumor microenvironment and/or stress‐inducing chemotherapeutic agents. This adaptation is driven mostly at the transcriptional level by oncogenic transcription factors, many of which have been shown to promote tumor progression in both the early and late stages across cancer types [1, 2]. One such transcription factor is nuclear factor erythroid 2‐related factor 2 (NRF2), which has been shown to play a critical role in mediating all the established hallmarks of cancer [3]. NRF2 target genes are involved in almost every aspect of metabolism, including maintaining redox homeostasis, mediating lipid, carbohydrate and iron processing, coordinating phase I, II and III xenobiotic metabolism, and controlling proteostasis [4]. Thus, determining how NRF2 and its downstream effectors promote tumor survival and resistance in different cancer contexts could facilitate the development of new treatment strategies.

Among the cancers associated with pathogenic activation of the NRF2 pathway is lung cancer, which is responsible for the highest percentage of cancer‐related deaths in both men and women in the United States each year. Non‐small cell lung cancer (NSCLC), which accounts for ~ 80–85% of all lung cancer cases, exhibits mutations, or copy number alterations in *NFE2L2*/NRF2 or its degradation machinery (*KEAP1, CUL3,* or *RBX1*) in > 30% of NSCLC cell lines and patient tissues [5, 6]. As such, many NSCLCs have been dubbed “NRF2‐addicted”, with constitutive activation of NRF2 mediating lung cancer progression and poor patient prognosis. Unfortunately, the discovery and development of NRF2‐specific inhibitors is an unmet challenge. However, indirect NRF2 inhibitors that inhibit protein translation have revealed the importance of NRF2 as a drug target [7]. Despite the difficult nature of targeting NRF2 directly, it is important to develop a mechanistic understanding of NRF2 target gene function and their potential as drug targets to kill cancers where NRF2 is overexpressed.

Another important regulator of cell growth and survival is the mTOR signaling cascade, which is a mediator of numerous cellular pathways, including amino acid, glucose, and fatty acid metabolism, as well as protein synthesis and the autophagy pathway [8]. Thus, as one might expect, dysregulation of mTOR and its downstream cascades have been shown to play a role in cancer development and progression, with the vast mTOR signaling cascade providing a host of viable therapeutic targets to treat different cancer types [9, 10]. mTOR exists in two different complexes, mTORC1 and mTORC2, each of which have their own complex components and upstream mediators that dictate their activation depending on the environmental cue received. Despite the upstream signal, an important component of mTOR function is its translocation to the lysosome, where it is anchored and activated by an intricate complex of lysosomal membrane‐bound proteins and cytosolic interacting partners that play an integral role in governing mTOR function [11].

In the case of amino acid sensing, there are several critical players that initiate the lysosomal recruitment of mTOR and its eventual phosphorylation/activation of downstream mediators of protein translation. Specifically, CASTOR1 and sestrin2 are l‐arginine and l‐leucine‐sensitive proteins, respectively, that inhibit GATOR1 and GATOR2‐dependent inhibition of the RagA/B and RagC/D heterodimeric complex. Rag proteins are normally anchored to the lysosomal membrane by the pentameric Ragulator complex; however, the presence of l‐arginine or l‐leucine prevents this inhibitory cascade, allowing the Rag‐Ragulator complex to recruit the cytosolic mTORC1 complex to the lysosome, where it is activated by the GTPase Rheb [11]. Once activated, mTOR phosphorylates downstream targets S6 kinase (S6K) and 4EBP1 to initiate protein translation. Importantly, while mTOR translocation to the lysosome is known to be necessary for its function, the specific trafficking machinery that mediates this process remains poorly understood. Normally, the Rab family of GTPases are mediators of vesicle trafficking and membrane fusion events. Proper Rab function requires geranylgeranylation, a lipid‐based modification regulated by geranylgeranyltransferases (GGTases) that allows insertion into target membranes. Rab activation is mediated by the Rab escort proteins, Rep1 and Rep2, which present their target Rab proteins to GGTases for geranylgeranylation [12].

Despite the importance of the Rep and Rab proteins in mediating vesicle tracking and complex anchoring to target membranes, the specific role of the Rep/Rab interaction in mediating mTOR trafficking has yet to be determined. Furthermore, whether mTOR activation can be dictated by NRF2, particularly in a lung cancer setting where both pathways are more active, is unknown. Here, we identified CHM‐like Rab escort protein (*CHML*/Rep2) as a novel target gene of NRF2 that mediates mTOR activation and is a critical mediator of NSCLC survival. Our findings demonstrate a previously unidentified intersection between the NRF2 and mTOR signaling cascades, two critical pathways critical for cell growth and survival.

## 2 Materials and methods

### 2.1 Chemicals, antibodies and reagents

l‐arginine (A8094), the primary antibodies against Rep2 (029628), LC3 (L7543) and ATG7 (A2856), as well as horseradish peroxidase‐conjugated secondary antibodies (goat anti‐rabbit, (A0545); goat anti‐mouse, (A9044)) were purchased from Sigma. The primary antibodies against GAPDH (sc‐32233), NQO1 (sc‐32793), NRF2 (sc‐13032), RAB7 (sc‐376362) and phospho‐S6 (sc‐293144) were purchased from Santa Cruz. LAMP1 (9091), mTOR (2983), phospho‐mTOR (5536), S6K (9202), phospho‐S6K (9234), and S6 (2217) antibodies were all obtained from Cell Signaling. The primary antibody against SQSTM1/p62 (89‐015‐843) was obtained from Abnova, and the SNAP29 antibody from Proteintech (12704‐1‐AP). *CHML* Flexitube siRNA constructs were purchased from Qiagen. The A549, H1299, A375 and MDA‐231 cell lines were all purchased from the American Type Culture Collection (ATCC). Dulbecco’s modified Eagle’s medium (DMEM; MT10014CV) was purchased from Corning. DMEM low glucose without amino acids was purchased from US Biological Life Sciences (D9800‐13). (FBS; S11150H) was purchased from Atlanta Biologicals. l‐glutamine (25030081) and penicillin‐streptomycin (15140722) were obtained from Gibco.

### 2.2 Biotinylated‐ARE pulldown

Biotin‐DNA pull‐down was performed as reported previously [13]. Briefly, cells were lysed in RIPA buffer containing 1 mm DTT, 1 mm phenylmethylsulfonyl fluoride (PMSF) and 1% protease inhibitor cocktail (Sigma‐Aldrich, St. Louis, MO, USA). The cell lysates were precleared with streptavidin beads and incubated with 2 μg biotinylated DNA probes that spanned the ARE‐containing sequences in the promoter region of *CHML*. The DNA–protein complexes were pulled down by streptavidin beads, and complexes were washed 3 times, resolved on an SDS/PAGE gel, and subjected to immunoblot analysis. The sequences of the 41‐bp biotinylated DNA probes used are as follows:

*CHML*‐wt ARE ‐ 5’‐GCTTTATAAGGGCAATGACTCAGCAATGAAGAATGAATAGG‐3’

*CHML*‐mt ARE ‐ 5’‐GCTTTATAAGGGCAAACTCTCACGAATGAAGAATGAATAGG‐3’

### 2.3 RT‐PCR

Total mRNA was extracted using TRIzol (ThermoFisher, Waltham, MA, USA) according to the manufacturer’s instructions. cDNA was then synthesized using 2 μg of mRNA and a Transcriptor first‐strand cDNA synthesis kit (Promega, Madison, WI, USA). Real‐time qPCR was then performed as previously described [14]. GAPDH was used for qPCR normalization, and all experiments were measured in triplicate. Primer sequences (5’ to 3’) are as follows:

*CHML* F – AGGTTTGCCCGAATCCATCC

*CHML* R – TTCATGGATCAGGTCCTGCC

*MTOR* F – GTCTCGGCAACTTGACCATC

*MTOR* R – AAATGCTGCATGTGCTGGAA

*S6KB1* F – CGACAGCCCAGATGACTCAA

*S6KB1* R – ATTTGACTGGGCTGACAGGT

*S6* F – TTGAAGTGGACGATGAACGC

*S6* R – TTGTTTGTCGTTCCCACCAC

*GADPH* F – CTGACTTCAACAGCGACACC

*GADPH* R – TGCTGTAGCCAAATTCGTTGT

### 2.4 Chromatin immunoprecipitation (ChIP)‐PCR

A ChIP assay was performed according to the manufacturer's instructions (EZ‐CHIP^TM^, Merck, Germany). Briefly, A549 WT or NRF2 KO cells were treated with 1% formaldehyde in DMEM for 10 min to cross‐link DNA‐protein complexes. The cells were then lysed using SDS lysis buffer containing 1 mm phenylmethylsulfonyl fluoride (PMSF) and 1% protease inhibitor cocktail (Sigma). Solubilized chromatin was then incubated with anti‐NRF2 antibody (Santa Cruz Biotechnology, Dallas, TX, USA) or normal rabbit IgG (Santa Cruz Biotechnology, Dallas, TX, USA) for 16 h at 4 °C with rotation, and DNA‐protein complexes were pulled down using Protein G‐agarose beads (Sigma, St. Louis, MO, USA). DNA from the immunoprecipitated complexes and total chromatin input were extracted via ethanol precipitation, and 1 μL of purified DNA was amplified and run on an agarose gel. Primers were as follows:

*CHML*‐ARE‐F‐TGTTTGTTCTCCCAACACGA

*CHML*‐ARE‐R‐TGTCGAAAGTGTTTTCTGTGTCT

### 2.5 Dual luciferase assay

For the dual luciferase assay, a 41 bp portion of the human *CHML* promoter containing the putative ARE sequence (or its mutated counterpart) was amplified by PCR, and then cloned into the pGL4.22 luciferase vector (Promega, Madison, WI, USA). Next, H1299 WT or Keap1 KO (KKO) cells were co‐transfected with 1 µg of a plasmid encoding a *Firefly* luciferase under the control of either a WT or MT‐*CHML* ARE‐driven promoter, as well as 1 µg of a *Renilla* luciferase plasmid under the control of a universal promoter as an internal control. Luciferase activity was measured using the dual luciferase reporter assay system (Promega). For relative luciferase activity analysis, the value of Firefly luciferase was normalized to the value of Renilla luciferase.

### 2.6 Immunoblot analysis

To detect changes in protein expression, A549 WT, A549 NRF2 KO, H1299 WT, H1299 KKO, BEAS‐2B, A375 and MDA‐231 cells were seeded in 6 well plates and 24 h later were either left untreated or treated with the indicated concentrations of sulforaphane, brusatol or l‐arginine for the indicated time points. Following treatment, cells were washed twice with 1X PBS and harvested in 1X Laemmli buffer (31.25 mm Tris‐Cl, 1.5% SDS, 5% glycerol, 5% β‐mercaptoethanol and 0.05% bromophenol blue), and boiled for 10 min. Cell lysates were then resolved by SDS/PAGE and subjected to immunoblot analysis with the indicated antibodies. All immunoblot images were taken using the Azure 600 imaging system (Azure Biosystems, Dublin, CA, USA) and analyzed using ImageJ 1.51s (NIH).

### 2.7 Immunohistochemistry

Slides containing fixed normal (*N* = 14) and lung adenocarcinoma (*N* = 140) tissue (BioMax) were processed for NRF2 and Rep2 expression as described previously [15]. Briefly, sodium citrate buffer (0.01 m, pH = 6.0) was used for antigen retrieval, and endogenous peroxidase activity was blocked using 0.3% H_2_O_2_. Slides were blocked with 5% BSA for 30 min and then incubated with primary antibodies against NRF2 (1 : 100), SLC7A11 (1 : 100), or Rep2 (1 : 50) overnight at 4 °C. The next day, slides were washed with 1X PBS and stained using the Envision + System‐HRP kit (Dako) as per the manufacturer’s instructions. Images were taken on a Nikon Eclipse 50i microscope using the NIS Elements software v4.0.

### 2.8 siRNA mediated knockdown or exogenous overexpression of CHML

To knockdown *CHML*/Rep2, A549 WT cells were seeded in a 6‐well plate following transfection with 5 nm of non‐targeted (NT) or 5 nm of *CHML* siRNA using Qiagen Hiperfect as per the manufacturer’s instructions. Four siRNA constructs against each target of interest were obtained from Qiagen (Flexitube), and the construct that obtained the maximum knockdown was utilized for further study. For the overexpression studies, A549 WT cells were transfected with either 1 µg of an empty vector or 1 µg of a *CHML* encoding plasmid (Origene; RC218675L3) for 24 h using Lipofectamine 3000 as per the manufacturer’s instructions. Following knockdown or overexpression, cells were left untreated, or treated with the indicated concentrations of sulforaphane, brusatol or l‐arginine for the indicated time points.

### 2.9 Cell viability and determination of percent confluence

Cell viability was determined using an MTT assay. Briefly, following siRNA‐mediated knockdown of Rep2, cells were incubated with 20 μL of MTT reagent (5 mg·mL^−1^) for 2 h. Following incubation, media was removed and isopropanol‐HCL was added and absorbance at 570 nm was measured using a SpectraMax iD5 Multi‐Mode microplate reader (Molecular Devices). Cell percent confluence was determined using the Incucyte ZOOM Live Cell Analysis system (Essen Biosciences).

### 2.10 Endogenous immunofluorescence

To determine if mTOR co‐localizes with LAMP1, endogenous immunofluorescence was utilized. Briefly, A549 WT cells were seeded in glass bottom D35 dishes. The next day, cells were amino acid starved for 30 min, and left untreated or treated for 15 min with 1 mm l‐arginine. Following treatment, cells were washed 3X with 1X PBS and fixed in ice cold methanol for 20 min. After fixation, cells were washed 3X with 1X PBS, incubated with primary antibody (1 : 1000 in 1X PBS with 10% FBS) at 4°C overnight, washed again and incubated with fluorescent secondary antibodies (1 : 2000; Alexa594‐anti Rabbit and Alexa488‐anti Mouse) for 1 h. Finally, cells were washed, mounted and imaged on a Zeiss Observer.Z1 microscope using the Slidebook 4.2.0.11 software (Intelligent Imaging Innovations, Inc.).

### 2.11 Transfection and live cell fluorescent imaging

For live cell imaging, A549 WT cells transfected with 1 µg of a *CHML* encoding plasmid (Origene) for 24 h or treated with *CHML* siRNA for 72 h were then transfected with 1 µg of the mRFP‐GFP‐LC3 tandem fluorescent reporter plasmid using Lipofectamine 3000 according to the manufacturer’s instructions. 24 h later, cells were amino acid starved for 30 min, and left untreated or treated for 15 min with 1 mm l‐arginine. Prior to imaging, cells were washed with 1X PBS and imaged in DMEM without phenol red. Images were taken on a Zeiss Observer.Z1 microscope using the slidebook 4.2.0.11 software (Intelligent Imaging Innovations, Inc.).

### 2.12 SILAC determination of protein synthesis

To determine changes in protein synthesis, A549 WT cells were seeded in 6 well plates and treated with non‐targeted (NT) or *CHML* siRNA for 72 h (*N* = 6 per condition). Following knockdown, cells were placed in low glucose DMEM containing 0.1 g·L^−1 13^C_6_^15^N_2_ Lys and 0.1 g·L^−1 13^C_6_^15^N_4_ Arg for 24 h. Next, cells were washed once with ice‐cold 1X PBS and pelleted. Cells were lysed in 150 µL of a buffer containing 150 mm NaCl, 50 mm HEPES, pH 7.4, 1% Igepal, and protease and phosphatase inhibitor cocktails (1 : 500 v/v, Sigma Aldrich, St. Louis, MO). Cells were sonicated and insoluble debris was removed via centrifugation at 14 000 ***g*** for 10 min at 4 °C. Soluble protein was precipitated in 300 µL of acetone for 24 h at −80 °C. Precipitated protein was pelleted via centrifugation at 14 000 ***g*** for 10 min at 4 °C and acetone was removed and pellets were allowed to air dry. Protein was then digested in 50 µL of 50 mm NH_4_HCO_3_ (pH 8.0) with sequencing grade trypsin (~ 1 : 100 w/w, Promega) for 6 h at 37 °C. Trypsin was denatured via boiling at 95 °C for 10 min and samples were allowed to cool to room temperature. Aminopeptidase (15 µg in 10 µL, Millipore) was then added to each sample and incubated overnight at 37 °C. Aminopeptidase was denatured via heating at 95 °C for 10 min and samples were cooled to room temperature. 15 µL of heptafluorobutyric acid (HFBA, 1 : 1 in ddH_2_O) and water (15 μL) was added to each sample. Debris was removed via centrifugation at 14 000 ***g*** for 10 min and 10 µL of the clarified supernatant was chromatographed using a Shimadzu LC system equipped with a 150 × 2.1 mm, 3.5 µm particle diameter Eclipse XDB‐C8 column (Agilent, Santa Clara, CA) at a flow rate of 0.35 mL·min^−1^. Mobile phase A : 10 mm HFBA in water; mobile phase B: 10 mm HFBA in ACN. The following gradient was used: 0.5 min, 5% B; 8 min, 50% B; 8.5 min, 80% B; 9 min 80% B; 9.5 min, 5% B. The column was equilibrated for 5 min at 5% B between runs. MRM was conducted in positive ion mode using an AB SCIEX 6500+ QTRAP. Table 1 indicates the parameters used, including the transition values and collision energies (CE), and data is presented as % heavy isotope incorporated [16].

**Table 1:** MRM Transitions and CE values for indicated analytes.

Species	Q1 (*m/z*)	Q3 (*m/z*)	Dwell Time (msec)	CE (V)
Lys	147.0	84.0	75	25
^13^C_6_^15^N_2_ Lys	155.0	90.0	75	25
Arg	175.0	70.0	75	51
^13^C_6_^15^N_4_ Arg	185.0	75.0	75	51

### 2.13 Statistical analysis

All results are expressed as mean ± standard deviation (SD) from three independent experiments. Sample size (*n*) is indicated in the respective figure legends. An unpaired student’s t‐test or one‐way ANOVA was used to determine statistical significance, with *P* < 0.05 being considered significant.

## 3 Results

### 3.1 CHML is an NRF2 target gene

**Fig. 1:**
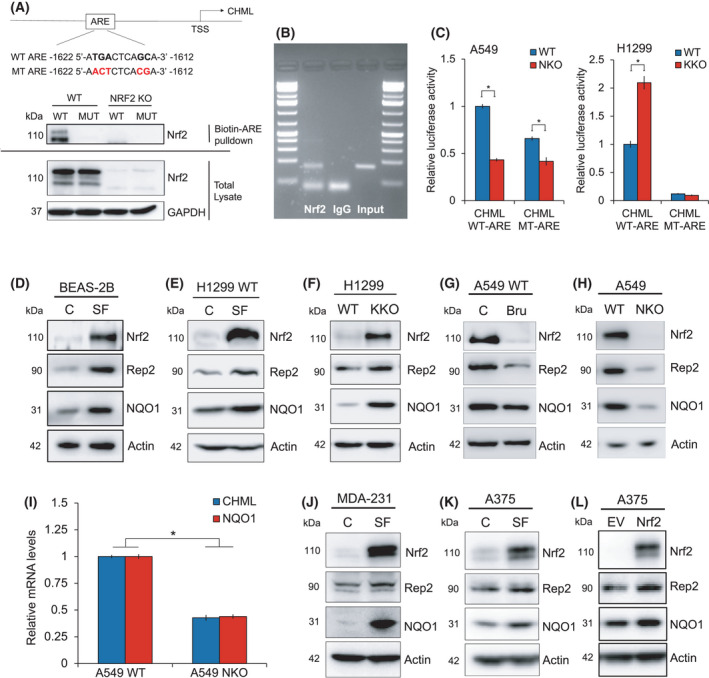
*CHML/*Rep2 is an NRF2 target gene. (A) Sequence and upstream location of putative *CHML* antioxidant response element (ARE; ^−1622^ATGACTCAGCA^‐1612^). Biotinylated wild type (WT; ATGACTCAGCA) or mutant (MT; AACTCTCACGA) ARE‐containing oligonucleotides were incubated with A549 WT or NRF2 knockout (KO) cell lysate and ARE‐bound proteins were pulled down using streptavidin beads. NRF2 protein levels were assessed via immunoblot analysis. GAPDH was used as an internal loading control. (B) ChIP‐PCR of NRF2‐bound DNA immunoprecipitated from A549 WT cells using an anti‐NRF2 antibody. A region of the *CHML* promoter containing the putative ARE was amplified and compared to an IgG control. (C) A549 WT and NRF2 KO or H1299 WT and KEAP1 KO cells were co‐transfected with 1 µg of a plasmid encoding a *Firefly* luciferase under the control of either a WT or MT‐*CHML* ARE‐driven promoter, as well as 1 µg of a *Renilla* luciferase plasmid under the control of a universal promoter as an internal control and subjected to a dual luciferase activity assay. Data = mean ± SD. *n* = 3 per group. *P < 0.05 compared to WT control. Unpaired student’s t‐test. (D‐H) Immunoblot analysis of NRF2, Rep2, and NQO1 protein levels in BEAS‐2B or H1299 WT cells treated with 5 µm sulforaphane (SF) for 16 h (D‐E), H1299 WT vs. KEAP1 KO cells (F), A549 WT cells treated for 16 h with 40 nm Brusatol (G), or A549 WT vs. NRF2 KO cells (H). (I) *CHML* mRNA levels in A549 WT vs. NRF2 KO cells. Data = mean ± SD. *n* = 3 per group. *P < 0.05 compared to A549 WT control. Unpaired student’s t‐test. (J‐L) Immunoblot analysis of NRF2, Rep2, and NQO1 protein levels in MDA‐231 and A375 cells treated with SF for 16 h (J‐K) or A375 cells transfected with 1 µg of an NRF2 plasmid for 24 h (L). All groups for immunoblot analysis were performed in triplicate, and experiments were repeated two times to ensure validity of results.

Analysis of DNA microarray data from NRF2 wildtype (WT) and NRF2 knockout (NKO) immortalized bronchial epithelial (BEAS‐2B) cells indicated that *CHML* levels were lower in BEAS‐2B NKO cells compared to WT (data not shown), suggesting it may be transcriptionally regulated by NRF2. *In* *silico* analysis of the *CHML* gene regulatory DNA sequence revealed several putative antioxidant response elements (AREs), including one within 2 kb of the transcriptional start site (^−1622^ATGACTCAGCA^‐1612^) (Fig. 1A) that was confirmed to be a functional ARE. NRF2‐ARE binding was confirmed by streptavidin‐mediated pulldown of the wildtype biotinylated ARE (WT–ATGACTCAGCA) but not the mutated biotinylated ARE (MUT–AACTCTCACGA) when incubated with protein lysate from A549 WT cells. As expected, NRF2 was not detected in the pulldown when the wildtype biotinylated ARE or the mutated biotinylated ARE were incubated with A549 NKO cell lysate (Fig. 1A). Endogenous NRF2 binding to the region of the *CHML* promoter containing the ARE was confirmed by ChIP‐PCR (Fig. 1B). Next, a 41 bp ARE‐bearing DNA sequence from *CHML* was cloned into the promoter of a firefly luciferase construct, and ARE functionality was determined by dual luciferase assay. H1299 KEAP1 knockout cells (KKO), which have higher NRF2 due to loss of KEAP1‐dependent degradation of NRF2, exhibited higher ARE‐driven luciferase activity than their WT counterparts (Fig. 1C). ARE‐driven luciferase activity was also decreased in A549 NKO cells compared to WT, and luciferase activity was significantly lower in the presence of the MUT‐ARE construct across all cell types (Fig. 1C). These results clearly demonstrate that *CHML* is an NRF2 target gene. As mentioned above, *CHML* encodes the protein product CHM‐like Rab escort protein (Rep2). To determine if NRF2 regulates *CHML*/Rep2 levels in lung cells, NRF2 was either genetically or pharmacologically manipulated in BEAS‐2B (normal lung epithelial cell), H1299 (NSCLC) or A549 (NSCLC) cells and Rep2 protein levels were assessed (Fig. 1D‐H). Treatment with the well‐established NRF2 inducer sulforaphane (SF) increased the levels of Rep2, as well as NQO1, a known NRF2 target gene, in both BEAS‐2B and H1299 cells (Fig. 1D‐E). Consistent with the luciferase assay, H1299 Keap1 KO cells had higher basal levels of NRF2, as well as Rep2 and NQO1 (Fig. 1F). A549 cells, which have a mutation in *KEAP1* that results in constitutive activation of NRF2, had significantly less NRF2, Rep2 and NQO1 following brusatol (Bru; Nrf2 inhibitor) treatment or genetic ablation of *NFE2L2*/NRF2, respectively (Fig. 1G‐H). NRF2 knockout resulted in a significant decrease in *CHML* mRNA levels (Fig. 1I). Finally, NRF2‐dependent regulation of *CHML*/Rep2 was also verified in other cancer cell lines, as MDA‐231 and A375 cells treated with SF, as well as A375 cells transfected with an NRF2 plasmid, all exhibited higher Rep2 levels compared to their relative controls (Fig. 1J‐L). These findings indicate that *CHML*/Rep2 is an NRF2 target gene, and that NRF2‐mediated *CHML* expression is not specific to lung cell types.

### 3.2 Increased expression of CHML/Rep2 correlates with decreased patient survival and increased expression of NFE2L2 and its target genes in human lung cancer patient tissues

**Fig. 2:**
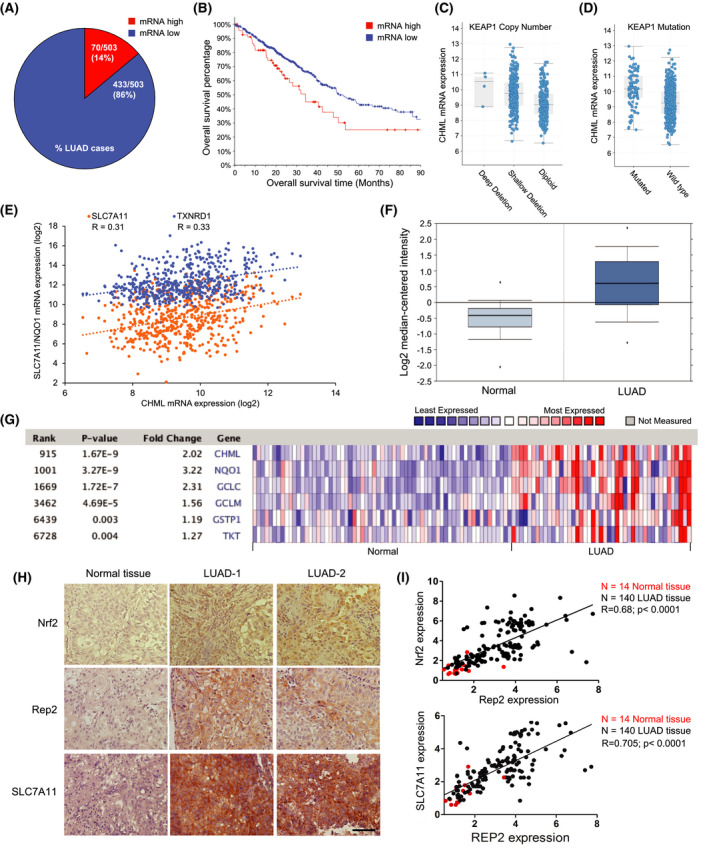
Increased expression of *CHML/*Rep2 correlates with decreased survival and increased expression of *NFE2L2* and its target genes in lung adenocarcinoma patients. TCGA analysis of (A) % of lung adenoma carcinoma cases (LUAD) exhibiting high *CHML* mRNA levels, (B) Patient survival based on *CHML* mRNA expression levels, and (C‐D) *KEAP1* copy number and mutation status compared to *CHML* mRNA levels from a lung adenocarcinoma patient cohort (*n* = 503). (E) Comparison of mRNA expression levels of *SLC7A11* and *TXNRD1* versus *CHML* from the same TCGA pan‐cancer atlas cohort. (F‐G) Curated Oncomine data indicating *CHML* mRNA levels in lung adenocarcinoma (*n* = 91) vs normal (*n* = 65) tissue compared to other well‐established NRF2 target genes. Red = higher expression, blue = lower expression. Genes are ranked based on significance of fold change compared to other genes. (H‐I) Immunohistochemistry analysis and correlation plot of NRF2/SLC7A11 levels and Rep2 expression in normal vs. lung adenocarcinoma (LUAD) patient tissues. Red dots = normal tissue (*n* = 14); Black dots = LUAD patient tissue (*n* = 140). *P* < 0.0001. One‐way ANOVA. Scale bar = 100 μm.

To determine the possible translational relevance of *CHML* in NSCLC patients, patient data from The Cancer Genome Atlas (TCGA) and Oncomine databases were analyzed. Lung adenocarcinoma (LUAD) patient data from the Pan‐Cancer Atlas indicated that ~ 14% (70/503) of patients exhibited high *CHML* mRNA levels, which correlated with a decrease in overall time of survival (Fig. 2A‐B). A more detailed analysis of how *CHML* levels coincided with NRF2 status indicated that loss of *KEAP1* heterozygosity/homozygosity (shallow/deep deletion) or *KEAP1* mutation, both of which are known to increase NRF2 levels in LUAD patients, correlated with an increase in *CHML* expression (Fig. 2C‐D). Furthermore, *CHML* expression was positively correlated with the expression of other known NRF2 target genes, including *TXNRD1* and *SLC7A11* (Fig. 2E). Further analysis of data from a study by Hou et al. curated by the Oncomine™ Platform also showed that *CHML* expression is significantly higher in LUAD patient lung tissue compared to normal lung, and that like the TCGA patient data, *CHML* expression correlates with that of other established NRF2 target genes [17] (Fig. 2F‐G). Finally, our own immunohistochemistry (IHC) analysis of 14 normal lung tissues compared to 140 LUAD tumor tissues also demonstrated higher levels of NRF2, SLC7A11 and Rep2 in LUAD lung tissue compared to normal controls, in addition to a strong correlation between Rep2 and SLC7A11 expression and NRF2 protein levels (Fig. 2H‐I). These data indicate that *CHML*/Rep2 expression is increased in NSCLC/LUAD patient tumors where NRF2 is high.

### 3.3 Knockdown of CHML decreases NSCLC cell proliferation

**Fig. 3:**
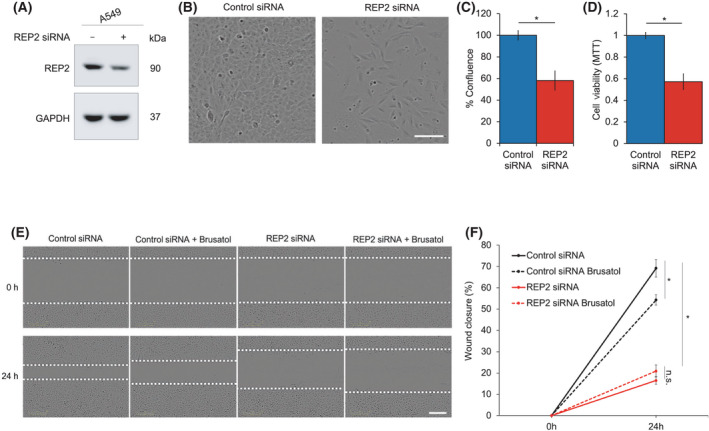
*CHML/*Rep2 knockdown decreases A549 proliferation and migration. (A) Immunoblot analysis of Rep2 protein levels in A549 WT cells following treatment with 5 nm of either NT or *CHML* siRNA for 72 h. (B) Representative images and (C) quantification of percent confluence (indicator of cell proliferation) following knockdown. Scale bar = 100 μm. (D) MTT assay for cell viability of A549 cells transfected with 5 nm of either NT or *CHML* siRNA for 24 h. (E) Representative images of a scratch assay (indicator of cell migration) following Rep2 knockdown and Bru treatment. Scale bar = 200 μm. (F) Quantification of % wound closure over the 24 h brusatol treatment period from (E). Data = mean ± SD. *n* = 5 per group. *P < 0.05 compared to control siRNA group. Unpaired student’s t‐test. All experiments were repeated two times to ensure validity of results.

Next, the importance of *CHML*/Rep2 in mediating A549 WT cell growth and migration was assessed using an siRNA‐mediated knockdown approach. First, successful knockdown of Rep2 was confirmed by immunoblot analysis (Fig. 3A). Following knockdown, the cell percent confluence was assessed as an indicator of changes in proliferation. Interestingly, knockdown of Rep2 resulted in an ~ 40% decrease in confluence, with control siRNA treated cells reaching ~ 100% compared to ~ 60% for the Rep2 siRNA group (Fig. 3B‐C). A similar decrease in cell number following knockdown was also observed using an MTT assay (Fig. 3D). Furthermore, the ability of Rep2 knockdown cells to migrate into open space, as determined by a scratch assay, was much more severely compromised than control siRNA treated cells (Fig. 3E‐F). Brusatol was only able to inhibit migration in control siRNA treated cells, but not Rep2 knockdown cells, presumably indicating that the compromised migration was largely derived from NRF2‐dependent Rep2 inhibition in control siRNA cells treated with brusatol. These results suggest that loss of *CHML*/Rep2 in A549 cells significantly inhibits their ability to proliferate and migrate.

### 3.4 Altering Rep2 levels in NSCLC cells does not alter the autophagy pathway

**Fig. 4:**
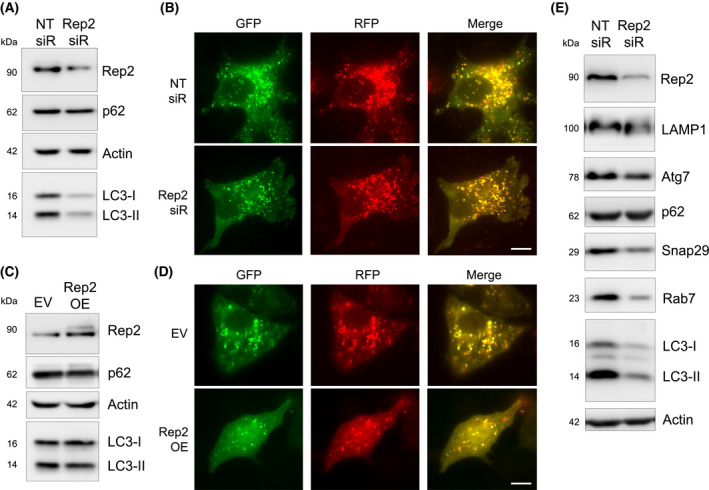
Knockdown of *CHML/*Rep2 decreases protein levels without affecting autophagy. (A) Immunoblot analysis of Rep2, p62, and LC3‐I/II protein levels in A549 cells treated with 5 nm of either NT or *CHML*/Rep2 siRNA for 72 h. (B) RFP‐GFP‐LC3 tandem fluorescence analysis of autophagy flux following siRNA knockdown same as (A). Yellow puncta = autophagosomes, red puncta = autolysosomes. Scale bar = 10 μm. (C) Immunoblot analysis of Rep2, p62, and LC3‐I/II protein levels in A549 cells transfected with 1 µg of either an empty vector (EV) or WT‐*CHML* (Rep2 OE) for 24 h. (D) RFP‐GFP‐LC3 tandem fluorescent analysis of autophagy flux following Rep2 overexpression same as (C). Scale bar = 10 μm. (E) Immunoblot analysis of Rep2, LAMP1, Atg7, p62, Snap29, Rab7, and LC3‐I/II protein levels following Rep2 knockdown for 72 h. All groups for immunoblot and immunofluorescence analysis were performed in triplicate, and experiments were repeated two times to ensure validity of results.

As Rep2 facilitates the geranylgeranylation of Rab proteins by geranylgeranyl transferases (GGTases), a key step in mediating Rab function, we hypothesized that Rep2 may play a role in key vesicle trafficking pathways such as the autophagy‐lysosomal pathway. While knockdown of Rep2 in A549 cells decreased LC3‐I/II levels compared to control siRNA treated cells, p62 levels were not significantly altered (Fig. 4A). To assess the effect of knockdown of Rep2 on autophagy flux, the mRFP‐GFP‐LC3 construct, where yellow puncta indicate autophagosomes and red puncta indicate autolysosome formation and successful completion of the autophagy pathway, was used. Intriguingly, while basal autophagy in A549 cells was high, which is to be expected of a NSCLC cell line, knockdown of Rep2 did not significantly alter the number of autophagosomes or autolysosomes present (Fig. 4B). Similarly, overexpression of Rep2 via introduction of a *CHML*/Rep2 encoding plasmid also had a minimal effect on LC3‐I/II protein levels and autophagy flux (Fig. 4C‐D). A survey of the protein levels of other key autophagy proteins revealed that knockdown of Rep2 significantly decreased the level of almost every protein tested (Fig. 4E). This implied that loss of Rep2 may decrease protein levels via a mechanism independent of altering autophagy flux.

### 3.5 Rep2 mediates mTOR signaling by controlling its translocation to the lysosome

**Fig. 5:**
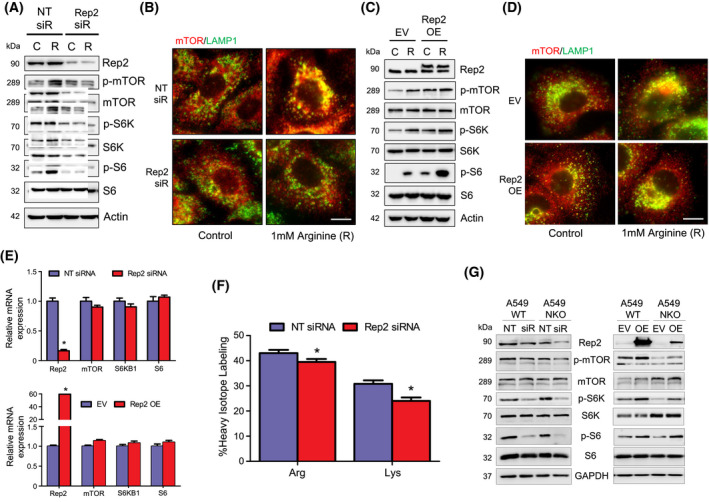
Knockdown of *CHML/*Rep2 decreases, whereas overexpression increases, mTOR activation. (A) Immunoblot analysis of phosphorylated mTOR (S2448), mTOR, phosphorylated S6K (S371), S6K, phosphorylated S6 (S235/236), and S6 protein levels in A549 cells treated with 5 nm NT or *CHML*/Rep2 siRNA for 72 h., then amino acid starved for 30 min., and left untreated or treated for 15 min. with 1 mm l‐arginine (R) to activate the mTOR pathway. (B) Endogenous immunofluorescence of mTOR (red) colocalization with LAMP1‐positive lysosomes (green) following knockdown and l‐arginine treatment same as (A). Scale bar = 10 μm. (C) Immunoblot analysis of the same proteins assessed in (A) in A549 WT cells transfected with 1 µg of either empty vector or WT‐*CHML* for 24 h, then amino acid starved for 30 min., and left untreated or treated for 15 min. with 1 mm l‐arginine (R). (D) Endogenous immunofluorescence of mTOR (red) colocalization with LAMP1‐positive lysosomes (green) following Rep2 overexpression and l‐arginine treatment same as (C). Scale bar = 10 μm. All groups for immunoblot and immunofluorescence analysis were performed in triplicate, and experiments were repeated two times to ensure validity of results. (E) RT‐PCR of *mTOR*, *S6KB*, *S6*, and *CHML* mRNA levels following Rep2 knockdown (72 h) or overexpression (24 h). Data = mean ± SD. *n* = 3 per group. *P < 0.05 compared to NT siRNA or EV group. Unpaired student’s t‐test. (F) SILAC determination of protein synthesis following knockdown with *CHML*/Rep2 siRNA and treatment with heavy arginine or heavy lysine for 24 h. Data = mean ± SD. *n* = 6 per group. *P < 0.05 compared to NT siRNA group. (G) Immunoblot analysis of mTOR pathway proteins in A549 WT vs. NRF2 KO cells treated with Rep2 siRNA or transfected with a WT‐*CHML* plasmid as described above. All groups for immunoblot analysis were performed in triplicate, and experiments were repeated two times to ensure validity of results.

Based on Rep2 knockdown decreasing the levels of numerous autophagy proteins independently of autophagic turnover, the role of Rep2 in mediating protein synthesis was tested. Protein translation is controlled by the mTORC1 complex, which senses changes in growth factor or nutrient status in the cell, and depending on the cue, phosphorylates different subsets of downstream targets. Similar to the observed decrease in autophagy machinery protein levels (Fig. 4E), the total protein levels of mTOR and its downstream target S6 kinase (S6K) were decreased in Rep2 siRNA treated cells (Fig. 5A). Furthermore, the phosphorylation of mTOR, S6K and ribosomal protein S6 were inhibited both basally and following treatment with l‐arginine (mTOR activator) in Rep2 siRNA treated cells (Fig. 5A). Since mTOR requires translocation to the lysosome to be fully activated, we hypothesized that loss of Rep2 might be affecting mTOR activation by preventing its trafficking to the lysosomal compartment. Indirect immunofluorescence of mTOR localization with the lysosomal marker LAMP1 revealed co‐localization following l‐arginine treatment in control siRNA, but not Rep2 siRNA treated cells (Fig. 5B). Contrastingly, overexpression of Rep2 increased mTOR pathway activity and translocation to the lysosome under basal and arginine stimulated conditions (Fig. 5C‐D). Decreasing or increasing Rep2 levels, by siRNA knockdown or plasmid overexpression respectively, did not affect mTOR, S6K, or S6 at the mRNA level (Fig. 5E). To determine if mTOR‐dependent protein synthesis was affected by knockdown of Rep2, incorporation of “heavy” arginine and lysine into proteins was assessed in Rep2 siRNA treated A549 WT cells. The ratio of heavy arginine/lysine vs. light arginine/lysine can be used as an indicator of newly translated proteins. As expected, knockdown of Rep2 resulted in a slight, but significant decrease in the incorporation of both heavy arginine and heavy lysine into proteins, indicating inhibition of protein synthesis (Fig. 5F). Finally, the NRF2‐Rep2‐mTOR axis was further confirmed in A549 NKO cells, as further depletion of *CHML*/Rep2 exacerbated the loss of mTOR signaling observed in A549 WT Rep2 siRNA treated cells, whereas overexpression of *CHML*/Rep2 partially restored mTOR pathway activation in A549 NKO cells (Fig. 5G). Thus, our results indicate that Rep2 regulates protein translation in NSCLC cells by controlling mTOR lysosomal translocation and activation.

## 4 Discussion

The ability to generate novel therapeutic strategies to treat cancer relies upon the identification of mechanisms of transformation, metabolic adaptation, and resistance. In the case of NSCLC, NRF2 represents a critical upstream driver of tumor progression and survival; however, directly targeting NRF2 remains elusive. One approach to address the challenge of developing NRF2‐based therapeutics is to find NRF2 target genes that mediate the oncogenic effects of constitutive NRF2 activation. Here, we identified *CHML*/Rep2 as a novel NRF2 target gene. *CHML* levels are high in lung adenocarcinoma patients, correlating not only with increased expression of other NRF2 target genes, but also poorer prognosis and decreased overall patient survival. Of note is that Rep2 can also be increased in an NRF2‐dependent manner in breast and melanoma cancer cell lines, indicating that *CHML* could play a role in promoting cancers other than lung cancer. This notion is further supported by a recent study that indicated increased *CHML* levels play a role in promoting the progression of hepatocellular carcinoma [18]. Importantly, knockdown of *CHML* in A549 NSCLC cells decreased their ability to proliferate and migrate. This implies that the high levels of *CHML* observed in lung adenocarcinoma patients could be a key driver of both early and late stages of tumor progression. Due to the role of *CHML*/Rep2 in facilitating Rab protein geranylgeranylation and subsequent function at their target vesicles/membranes, we originally hypothesized that Rep2 would play an important role in mediating the increased autophagy observed in NSCLC cell lines and tissues. However, autophagy flux was unaffected by knockdown or overexpression of Rep2, perhaps because knockdown, as opposed to full genetic ablation or pharmacological inhibition, still leaves sufficient Rep2 levels to maintain some level of autophagy flux.

**Fig. 6:**
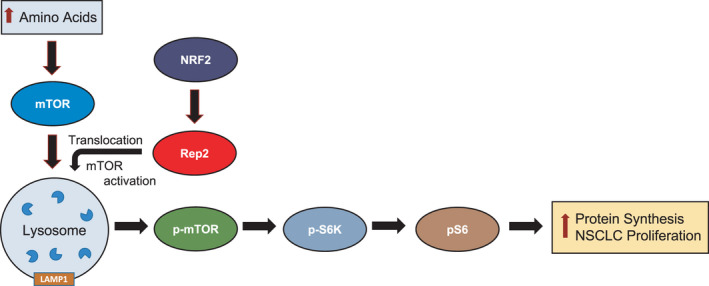
Summary Figure. Rep2 is a transcriptional target of NRF2 that mediates translocation of mTOR to the lysosomal membrane, where it is activated and initiates a downstream phosphorylation cascade that initiates protein translation. Loss of Rep2 decreases mTOR‐dependent protein synthesis leading to decreased proliferation in non‐small cell lung cancer (NSCLC) cells.

We also identified a functional relationship between *CHML*/Rep2 and the mTOR pathway (Fig. 6), with decreased expression inhibiting mTOR‐dependent protein synthesis, whereas overexpression increased mTOR activity. This would explain the decreased ability of A549 cells to proliferate when Rep2 is knocked down, as a critical energy sensing regulator of cellular homeostasis and protein translation is not functioning at full capacity. It is interesting that even though loss of Rep2 inhibits mTOR function, autophagy remained largely unaffected, as normally mTOR inhibition activates autophagic flux. This is presumably explained by the observed decrease in many autophagy proteins, including Atg7, which is critical for LC3 cleavage and subsequent autophagosome formation and initiation of the autophagy cascade. Regardless, A549 cells with Rep2 knocked down or overexpressed still maintained autophagy flux, indicating that the autophagy pathway can function independently of Rep2 levels, at least in this cell type. As opposed to significantly affecting autophagy, our data clearly indicated that Rep2 is necessary for mTOR to translocate to the lysosome, presumably through decreased interaction with its yet to be identified Rab interacting partner, or with the RagA/C or B/D GTPases, which as discussed above have been shown to mediate mTOR trafficking. Identification of the detailed mechanism by which Rep2 mediates mTOR translocation/activation, as well as a more detailed investigation of its relationship with autophagy flux will be performed in future studies.

## 5 Conclusion

Overall, this study generates one of the first links between NRF2 activation and mTOR lysosomal translocation/function. It is important to note that one study previously identified an ARE in the mTOR promoter region that could be bound by NRF2 [19]. However, while NRF2 overexpression in A549 WT cells increased mTOR levels, knockdown of NRF2 had no observable effect, which is inconsistent with mTOR being a direct NRF2 target gene. This is similar to our finding that mTOR mRNA levels in A549 NRF2 KO cells are similar to WT (data not shown). Importantly, they further go on to demonstrate that NRF2‐dependent regulation of mTOR occurs through the PI3K pathway and NF‐κB activation, which also indicates an indirect mechanism of regulation. We did not assess the PI3K pathway or NF‐κB in this study; however, we believe that our data firmly supports a role for Rep2 in mediating mTOR function. Despite these differences, the mTOR and PI3K pathways are closely linked, which could infer that these mechanisms may somehow overlap. Thus, further investigation of NRF2‐mTOR regulation and cross‐talk with other signaling cascades represents an interesting area of future research.

While there are no current compounds or drugs that target the Rep proteins, there are some drugs that target GGTases, which would achieve a similar effect to Rep2 inhibition, that have shown clinical promise [20, 21]. As such, future endeavors to not only target Rep2‐dependent activation of mTOR in NSCLC but also determine the relevance of this mechanism in other cancer types, including cross‐talk of the NRF2‐Rep2 axis with other key survival pathways, could provide a novel therapeutic approach to treat cancers that rely on increased NRF2 and mTOR function for survival.

## Conflict of interest

The authors have no conflicts of interest to declare.

### Peer review

The peer review history for this article is available at https://publons.com/publon/10.1002/1878‐0261.13194.

## Author contributions

MD, WD, AA, CJS, PL, NW, YW and NK performed the research and analyzed data. MD, JJG, AO, EC and DDZ designed the research strategy and wrote the paper.

## Data Availability

The data that support the findings of this study are available from the corresponding author (dzhang@pharmacy.arizona.edu) upon reasonable request.
